# Toll-Like Receptor Activation in Immunity vs. Tolerance in Autoimmune Diabetes

**DOI:** 10.3389/fimmu.2014.00119

**Published:** 2014-03-24

**Authors:** Elke Gülden, Li Wen

**Affiliations:** ^1^Section of Endocrinology, Yale University School of Medicine, New Haven, CT, USA

**Keywords:** Toll-like receptors, type 1 diabetes, immunity, tolerance, regulatory T-cells

## Introduction

### Toll-like receptors: Key receptors of the innate immune system

The human body constantly encounters a diverse spectrum of pathogens. To defend itself, a complex immune system has evolved consisting of two subdivisions: the innate and the adaptive immune systems. The innate immune system constitutes the so-called “first line of defense” and acts through highly conserved germ-line-encoded pattern recognition receptors (PRRs) (1). These receptors bind to common pathogen-associated molecular patterns (PAMPs), which are vital for the survival of the microorganisms and cannot be altered by mutations.

Pattern recognition receptors were first discovered in the fruit fly *Drosophila melanogaster*. The PRRs in *Drosophila*, named Toll, play an important role in the recognition of microorganisms such as fungi as well as coordinating embryonic development of the dorso-ventral axis.

Homologs of Toll in vertebrates are called Toll-like receptors (TLRs). The first TLR in humans was described in 1997 (2). To date, another 9 TLRs have been identified in humans and 13 in mice. Among these, only murine TLR10 is non-functional due to retrovirus insertion (3). Except for TLRs 3, 7, 8, and 9, which are expressed intracellularly, TLRs are located on the surface of innate and adaptive immune cells as well as on non-immune cells such as muscle cells, epithelial cells, adipocytes, and pancreatic beta cells (1). Cell-surface TLRs detect exogenous lipids, lipoproteins, and proteins from microbes; intracellular TLRs recognize bacterial and viral nucleic acids (3). Recognition of their cognate ligand triggers a complex signaling cascade that ultimately results in the induction of various pro-inflammatory chemokines and cytokines and the activation of the adaptive immune system.

However, the activation of TLRs and the subsequent induction of inflammatory immune responses are not always beneficial to the host. TLRs are involved in the pathogenesis of various autoimmune and non-infective inflammatory diseases such as systemic lupus erythematosus, multiple sclerosis, arteriosclerosis, inflammatory bowel disease, diabetes, allergy, and cancer (4, 5). Their activation can either attenuate or boost the course of disease by inducing tolerance or triggering autoreactivity, respectively.

The role of the TLRs in the pathogenesis of autoimmune-mediated diabetes, also referred to as type 1 diabetes (T1D), has been extensively studied (Table 1). T1D is a T-cell-mediated metabolic disorder with progressive destruction of insulin-producing pancreatic β cells (6). During the course of disease development, diabetogenic T-cells, macrophages, and dendritic cells will infiltrate the pancreatic islets and cause islet inflammation and eventually β cell loss.

**Table 1:** **TLR-related studies in the field of T1D**.

TLR/adaptor protein	TLR/adaptor protein		Effect on the course of T1D			Reference
	deficiency/blockade	sufficiency	attenuating/protective	promoting/boosting	dispensable	
TLR2		+	+			Filippi et al. (7)
TLR2	+	+		+		Kim et al. (8)
TLR2	+				+	Wen et al. (9)
TLR2	+		+			Devaraj et al. (10)
TLR2		+	+			Karumuthil-Melethil et al. (11)
TLR2	+			+		Al Shamsi et al. (12)
TLR2	+		+			Kim et al. (13)
TLR2, 3, 4, 7		+	+			Aumeunier et al. (14)
TLR3	+				+	Fallarino et al. (15)
TLR3	+				+	Wong et al. (16)
TLR4	+			+		Gülden et al. (17)
TLR4	+				+	Wen et al. (9)
TLR4	+		+			Devaraj et al. (18)
TLR4		+		+		Li et al. (19)
TLR7		+		+		Lee et al. (20)
TLR9	+		+			Zhang et al. (21)
TLR9	+		+			Tai et al. (22)
TLR9	+			+		Fallarino et al. (15)
TLR9		+		+		Zipris et al. (23)
TLR9	+		+			Wong et al. (16)
MyD88	+		+			Wen et al. (9)

*The table displays only studies that addressed a TLR directly. The studies that used TLR ligands but did not directly demonstrate the role of a specific TLR are not listed. ‘+’ denotes the composition (TLR or adaptor protein investigated; usage of wild type or knockout mice) and outcome of the study*.

This opinion letter focuses on the beneficial and detrimental aspects of TLR induction in the course of T1D development.

## TLR and Their Role in Autoimmune-Mediated Type 1 Diabetes

### TLR-mediated initial events in the induction of islet-directed immune responses

One of the major functions of the immune system is to distinguish self from non-self in order to fight invaders (pathogens) while sparing endogenous structures. PAMPs, the ligands of TLRs, are conserved molecular patterns that are exclusively expressed by pathogens. Therefore, the innate immune system is able to distinguish self and non-self. This beneficial “discrimination” is further enhanced by the adaptive immune system, in which most of the self-reactive T- and B-cells are deleted by central tolerance through negative selection. However, increasing evidence suggests that TLRs also recognize endogenous molecules including self-DNA released by injured or dying cells. These endogenous molecules act as danger signals and are therefore called “danger-associated molecular patterns (DAMPs).” The exposure of DAMPs by necrotic cells is considered to be a potential trigger of autoimmune diseases such as T1D (24). Studies have shown a defective clearance of dying cells in the NOD mouse (25), currently the best characterized model of human T1D (26). Due to the diabetes-prone genetic background, this defect might essentially contribute to the induction of autoimmunity in these mice. The accumulation of apoptotic β cells, which may undergo secondary necrosis (so-called “late state apoptosis”), could result in the activation of antigen-presenting cells (APC) via TLR engagement by released endogenous molecules and thereby contributing to the induction of diabetogenic T-cells (8, 27). Diabetogenic T-cells are then recruited to the pancreatic islets by chemokines like CCL2 (28, 29), CCL5, CXCL9, and CXCL10 (30) that can be released from β cells upon TLR ligation.

Consistent with this hypothesis is the fact that TLR2 induces apoptosis (31) and promotes diabetes in a streptozotocin-induced diabetic model following activation via the synthetic ligand Pam3CSK4 (12). The observation of delayed or reduced diabetes onset in TLR9-deficient mice further supports the theory (16, 21, 22). However, in a chemically induced diabetes mouse model, Fallarino et al. found that TLR9-deficient C57BL/6 mice were more susceptible to diabetes induction (15). This opposing finding is most likely due to the different choice of animal model. Other investigators have used the NOD mouse model (16, 21, 22) whereas Fallarino and colleagues induced diabetes by injections of β cell-toxic drug streptozotocin to C57BL/6 mice.

### Disease protection vs. disease induction

As key receptors of the innate immune system, TLRs trigger inflammatory immune responses upon binding of cognate ligands. On a predisposed genetic background, this event might initiate islet inflammation followed by progressive β cell destruction and finally overt T1D.

However, TLR activation is not necessarily causative for T1D development. T1D-related studies summarized in Table 1, reveal either a protective or detrimental effect on the induction of islet-directed autoimmunity. One reason for this dichotomy might be the point in time during the prediabetic phase when TLR activation is induced. Moreover, the presence or absence of β cell antigens and/or endogenous DAMPs released by late state apoptotic β cells possibly plays a critical role. Studies showed that activation of TLR2 (8), TLR3 (32), or TLR9 (33) by their cognate ligands in the presence of β cell antigens or DAMPs give rise to T1D development, whereas TLR stimulation in the absence of β cell antigens results in tolerogenic immune responses (11, 34–36). Observations by Filippi et al. suggest that the reason for this outcome might be the capacity of immunostimulatory factors to augment immune regulation (7).

### The role of TLRs in modulating Treg functions

Regulatory T-cells (Treg) are TLR-expressing adaptive immune cells which control immune responses in order to prevent aberrant immune reactions which could be harmful (37). In the presence of β cell antigens, TLR2 signaling could, while inducing a pro-inflammatory immune response, simultaneously promote suppressive Treg functions.

Dasgupta et al. demonstrated that engagement of TLR2 reverses the suppressor function of conjunctiva Treg in rabbits (38). In mice, in some cases, TLR2 stimulation resulted in a temporary abrogation of the regulatory capacity of Treg (39, 40), while other studies reported that the Treg function was either unchanged (41) or improved (7, 11). On the other hand, studies by other investigators showed a decreased number of Tregs following TLR2 stimulation (12).

One of the explanations for these contrary findings might be the different concentrations of the TLR ligand used in their experimental systems. It has been reported that the concentration of TLR ligands influences the regulatory activity of Tregs (40, 42, 43). For example, Zanin-Zhorov and colleagues showed that low doses of the TLR2 ligand, Hsp60, resulted in enhanced suppression without increased Treg proliferation (42, 43).

Studies by Round and Mazmanian investigating the immunomodulatory effect of polysaccharide A (PSA), a microbial molecule of the commensal bacterium *Bacteroides fragilis*, reveal that the PSA can signal directly on Treg cells via their TLR2 and promote immune tolerance (44, 45). It is possible that the effect may be accounted for by the anatomical site where TLR2 is engaged. Specifically in the intestine, TLR2 signaling induced by PSA is required for Treg induction and IL-10 expression.

TLR2 ligation does not merely exert influence on Treg; studies also reveal an effect on effector T-cells (46, 47). TLR2 signaling via Pam3Cys achieves resistance of T effector cells toward Tregs (47).

Besides TLR2, other TLRs also modulate Treg functions. TLR4 and TLR5 ligands are capable of boosting the suppressive function of Treg on T effector cells (48, 49). In line with these observations is the finding that TLR4-deficient NOD mice exhibit significantly accelerated diabetes development and impaired suppressive function of Tregs, although the frequency of Tregs remains unchanged (17).

Taken together, TLRs act as important modulators of Treg proliferation and function. Treg function can either be enhanced or attenuated depending on the concentration of TLR ligands and the anatomical site of TLR engagement.

### Tolerance induction by TLR signaling

As potent activators of inflammatory immune responses, the activation of TLRs must be tightly controlled since over-activation or loss of negative regulation can lead to detrimental or even life-threatening effects as seen in the condition of sepsis (50, 51). Repeated exposure to the same ligand can therefore result in hyporesponsiveness or tolerance through down-regulation of TLRs and simultaneous up-regulation of a negative feedback loop (50, 52).

In contrast to short term TLR stimulation, which results in initiation of immune responses, repeated exposure to TLR ligands might lead to the abrogation of inflammatory immune responses. In such a manner, TLR2 tolerance and inhibition of T1D development in NOD mice could be achieved by repeated administration of the TLR2 agonist Pam3CSK4 or zymosan (11, 13). Decreased T1D incidence was also accomplished following treatment with LPS, poly (I:C) (34), or CpG oligonucleotides (36).

As systemic chronic TLR stimulation suppresses the development of T1D in NOD mice (14, 53), it is possible that the absence of TLR stimulation might facilitate T1D development. This possibility is supported by the so-called hygiene hypothesis (54). The hygiene hypothesis coincides with the increase in allergy and autoimmune diseases over the past decades, possibly due to less exposure to microbial products as the hygiene standard has been significantly improved. Supporting the hygiene hypothesis is a recent study revealing that NOD mice deficient in the innate adaptor protein MyD88 are protected from diabetes development in non-germ-free conditions but the protection is abolished in germ-free conditions (9). Furthermore, introducing gut commensals into germ-free mice re-establishes the protection. This study supports the crucial role of environmental (TLR) stimuli in modulating the pathogenesis of diabetes through commensal bacteria.

## Concluding Remarks

The important role of TLRs in the pathogenesis of T1D manifests mainly in their ability to induce APC maturation and to produce inflammatory chemokines and cytokines. These two features will contribute to the priming of autoreactive T-cells, which cause islet cell destruction. However, TLR signaling can also induce immune tolerance that results in diabetes prevention depending on the genetic background and the environment.

Increasing evidence suggests that TLRs also express on tissue cells including pancreatic beta cells. The role of TLRs on islet beta cells is largely unknown. Due to the complexity of the T1D pathogenesis, there is still no cure or ultimate prevention from the disease development. TLRs are critical modulators of islet-directed immune responses and are, therefore, important targets for anti-diabetogenic therapies. However, many functions of TLRs and causal relations are still unknown. Many questions must be answered before we can generate novel and effective therapeutic approaches that target TLRs in treating T1D. Since therapeutic targeting of TLRs can also increase the susceptibility toward infections, safety and efficacy have to be thoroughly balanced when modulating TLRs.
